# Progress of the London's Experiment

**Published:** 1920-09-11

**Authors:** 


### The Hospital

The Workers' Newspaper of Administrative Medicine and Institutional Life, Administration, National Insurance and Health.

No. 1788, Vol. LXVIII. SATURDAY, SEPTEMBER 11, 1920. PRICE TWOPENCE

### PROGRESS OF THE LONDON'S EXPERIMENT.

The report to the quarterly court of governors of the London Hospital, which will be found at length on another page, describes the progress of the scheme for providing additional income through payments made by patients towards the cost of their maintenance; the information given is a valuable guide to other hospitals which are prospecting the same method of money-raising, for there must be a large number in that position.

With regard to this question of patients' payments, which is an entirely different thing from a system of wards for contributing patients, we have always felt that the hospitals of the provinces, with the exception of those to be found in very large cities, are, generally speaking, in quite a different position to the hospitals of the Metropolis. This is so because as a rule a provincial hospital has a district which supports it very liberally, and has a distinct claim upon it; while in London, in Manchester, in Liverpool, and other large centres, the claim of the district is far less insistent in respect of the general hospitals, and does not exist at all with regard to the special hospitals. Therefore the greater number of the hospitals of the great cities need hardly look further than to the condition of their own exchequers in seeking a decision, although it may be said in passing that not all the institutions will find there a sufficient financial reason for a departure from carrying on the work on the old lines; and, moreover, a number cannot make charges to patients at all without a complete alteration of their constitution. Amongst the latter class perhaps may be included the Royal Free Hospital, the Samaritan Free Hospital, and the Cancer Hospital. Very possibly such charities will elect to proceed as far as possible on the old lines.

It seems inevitable that the majority of the hospitals of large cities, however, will follow the lead of the London Hospital and adopt the paying principle, and in this it must be remembered they are treading the path which has been pursued by most of the medical charities of the colonies and foreign countries. What is chiefly the question of the moment is in what manner the principle will be applied, but even here no permanent decision can be arrived at until Parliament discloses its intentions with regard to Dr. Addison's Miscellaneous Powers Bill.

The committee of the London Hospital are quite alive to this point, for they express their opinion that the three major elements of the income of the future will be voluntary contributions, patients' payments, and State or Municipal aid. Naturally we are very glad that the sources of revenue are placed in the order named, and so long as they can be classified in this manner the voluntary principle must remain paramount. In fact, the result of Lord Knutsford's appeal gives great encouragement, and although the present financial position of the great East End hospital by no means dispels the cloud of anxiety that lowers over the general situation, it does not, in our opinion, justify the extreme feeling of pessimism which has in more than one instance caused hospital committees temporarily to lose their sense of proportion. Lord Knutsford asked his friends for support, and he received it in no inconsiderable measure, in fact, to the extent of over £50,000 in a very short period, although this success, we are reminded, may to some extent discount the quinquennial appeal of 1923. This warning does not alarm us, as we regard it simply as part of the general technique of the successful beggar, and we venture to think that the somewhat distant fortunes of 1923 may be left for the present to take care of themselves.

Of the £4 4s. a week, which is the approximate cost of the weekly maintenance of a bed at the London, £1 1s. is demanded from the patient, and we are told that the present result is additional revenue to the extent of about £20,000 a year. This contribution from the patient cannot be claimed seriously to make a breach in the voluntary principle. It barely covers the cost of food; the medical services, the nursing, the use of the extensive and expensive scientific equipment, the hundred other things which go to make up the complex organisation for which a modern hospital stands, still remain free gifts to the patient. Much more can be claimed from the occupant of a bed before it can be said that charity has ceased to be a large element in the bargain.

When we come to the third heading of revenue, as it is analysed by the committee of the London Hospital, we are dealing with an unknown quantity. We know what is likely to be received for the treatment of school children, and venereal and tuberculosis patients; but we do not know what may be received, should the powers of the Government departments be extended, from municipalities for the treatment of insured persons, nor can we see what is coming in on the educational side for the training of nurses and massage, electrical, and light operatives, apart from further State subsidy towards the cost of medical education.

For the moment we are passing through a period of readjustment. When we can see more clearly it is probable that the financial systems of the hospitals must undergo a change and procedure will be more on budgeting lines than in the past. Each hospital must take its probable forthcoming expenditure as divided between in-patients and out-patients and plan out definitely what it must aim to get towards the cost of each bed or each out-patient from the different sources of income, finding itself then in a position to make an assessment for the individual, for the municipality, or other responsible authority.

Before, however, any clear and satisfactory system of fianance can be applied, we must take in hand and deal with once for all the extremely difficult question of debt, not only debt in the sense of overdrafts at banks and other forms of cash indebtedness, but also as it is represented by deferred schemes of approved enlargement (which in most cases will be found to refer to altered conditions of nursing requirements) and much needed work in respect of redecoration and renewals generally. Debt in these larger senses will be found to be, we fear, much more considerable than is usually thought, and in our opinion is one of the chief factors of what is known as the "hospital crisis," although curiously enough the point is one to which hardly any real attention has been given. These problems must be dealt with in a very generous spirit, not only as regards public benefactions from individuals, corporations, and, if must be, from State departments, but as between the hospitals themselves, for we have noticed with regret a tendency, happily not very widespread, of hospital to criticise hospital and to throw out a charge that want of prudence has led to difficult positions and desperate remedies. Apart from the fact that it is impossible for one hospital to appreciate the inner workings of another with any amount of precision, it goes without saying that the governors of our voluntary institutions who are so kind as to give their time, experience, and influence, have always acted in good faith, and with a desire fairly to deal with those who tend them.

